# Non-Hematopoietic Cells in Lymph Nodes Drive Memory CD8 T Cell Inflation during Murine Cytomegalovirus Infection

**DOI:** 10.1371/journal.ppat.1002313

**Published:** 2011-10-27

**Authors:** Nicole Torti, Senta M. Walton, Thomas Brocker, Thomas Rülicke, Annette Oxenius

**Affiliations:** 1 Institute of Microbiology, ETH Zürich, Zürich, Switzerland; 2 Ludwig-Maximilians-University, Munich, Germany; 3 Institute of Laboratory Animal Science and Biomodels Austria, University of Veterinary Medicine Vienna, Vienna, Austria; Oregon Health Sciences University, United States of America

## Abstract

During human and murine cytomegalovirus (MCMV) infection an exceptionally large virus-specific CD8 T cell pool is maintained in the periphery lifelong. This anomalous response is only seen for specific subsets of MCMV-specific CD8 T cells which are referred to as 'inflationary T cells'. How memory CD8 T cell inflation is induced and maintained is unclear, though their activated phenotype strongly suggests an involvement of persistent antigen encounter during MCMV latency. To dissect the cellular and molecular requirements for memory CD8 T cell inflation, we have generated a transgenic mouse expressing an MHC class I-restricted T cell receptor specific for an immunodominant inflationary epitope of MCMV. Through a series of adoptive transfer experiments we found that memory inflation was completely dependent on antigen presentation by non-hematopoietic cells, which are also the predominant site of MCMV latency. In particular, non-hematopoietic cells selectively induced robust proliferation of inflationary CD8 T cells in lymph nodes, where a majority of the inflationary CD8 T cells exhibit a central-memory phenotype, but not in peripheral tissues, where terminally differentiated inflationary T cells accumulate. These results indicate that continuous restimulation of central memory CD8 T cells in the lymph nodes by infected non-hematopoietic cells ensures the maintenance of a functional effector CD8 T pool in the periphery, providing protection against viral reactivation events.

## Author Summary

Cytomegaloviruses (CMVs) infect the majority of the human population and persist lifelong via latency. CMV latency is thought to be a dynamic state, characterized by stochastic viral reactivation events coupled to CMV-derived antigen presentation. In support of this hypothesis is the exceptionally large CMV-specific CD8 T cell response which constitutes an integral part of immune surveillance of CMV reactivation. Conversely, it may also contribute to immune senescence as it significantly shapes the overall CD8 T cell pool in bias of CMV-specificity. In mice, only a subset of CMV-specific CD8 T cells, also called ‘inflationary CD8 T cells’, contribute to this large response. The mechanism leading to the selective accumulation and persistence of memory CD8 T cells during MCMV latency is largely unknown. Here, we unraveled the mechanisms of memory CD8 T cell inflation using a newly generated TCR transgenic mouse with specificity for an immunodominant inflationary MCMV epitope. We show that antigen presentation on non-hematopoietic cells is essential for memory inflation and that memory inflation in peripheral tissues is fueled by lymph node-resident central memory CD8 T cells, being locally reactivated by non-hematopoietic cells, inducing their local expansion and migration to peripheral tissues where they control viral reactivation events.

## Introduction

Memory CD8 T cells develop following primary encounter with an infectious agent and provide protection against subsequent infections. Depending on their phenotype and anatomical location, memory CD8 T cells have been categorized into central memory CD8 T cells (T_CM_) and effector-memory CD8 T cells (T_EM_) [1], [2]. T_CM_ localize to secondary lymphoid organs due to expression of the lymph nodes homing markers CD62L and CCR7, and are maintained by homeostatic proliferation responding to the cytokines IL-7 and IL-15 [3], [4]. Because of their self-renewal properties and their exquisite ability to proliferate and exert effector function upon re-encounter with the original pathogen, T_CM_ are able to provide long-term protective immunity. T_EM_, in contrast, reside predominantly in the periphery, usually at the site of primary infection, and possess a terminally differentiated phenotype, characterized by low expression of IL7Rα and CD62L and high expression of killer cell lectin-like receptor G1 (KLRG1). T_EM_ are believed to form the front line of defense against re-infections due to their immediate activation upon secondary infection at peripheral sites and due to their ready performance of effector functions [5], [6]. In absence of antigen, their long-term maintenance in the periphery is generally believed to be unstable. However, this notion has recently been challenged by the demonstration of long-term maintenance of effector memory cells in the skin, gut and brain in absence of detectable antigen persistence [7], [8], [9]. Studying the development and function of memory CD8 T cells should always take into account the nature of the infectious agent, as the duration of the initial antigen stimulation, the level of inflammation, the persistence of the pathogen, the cell tropism as well as the ability to interfere with the antigen presentation machinery of the host, are all factors influencing the development of the memory CD8 T cell response [10], [11], [12]. In particular, generation of memory responses are known to be highly perturbed during chronic or latent viral infections, where persistent antigen stimulation has often been shown to lead to functional impairment and antigen-dependent maintenance of CD8 T cells [13], [14], [15].

Infection with the β-herpes virus cytomegalovirus (CMV) leads to disseminated acute lytic replication which is controlled by various immune effector cells [16], [17], [18], [19], followed by life-long latency with presumably sporadic and low-level viral reactivation events [20], [21]. Thus, CMV infection allows studying memory CD8 T cell responses in the context of a latent/persistent viral infection. Both humans and mice are natural hosts of CMV and both elicit an impressively large virus-specific memory CD8 T cell response during latency which increases with time, a phenomenon referred to as ‘memory inflation’ [22], [23], [24], [25], [26]. Longitudinal analysis performed in mice revealed that upon infection with MCMV, two major kinetic patterns of CD8 T cell responses emerge: the majority of CD8 T cells, referred to as ‘conventional CD8 T cells’, undergo expansion during the acute phase of infection followed by rapid contraction, eventually resulting in low numbers of T_CM_ cells which are stably maintained during latency by homeostatic proliferation. In contrast, so called ‘inflationary CD8 T cells’, continue to expand even after control of acute infection, and are maintained at high percentages in the absence of overt viral replication [27], [28]. However, these cells display a T_EM_ phenotype, strongly suggesting that their accumulation and maintenance is driven by viral antigen [27], [28], [29]. Therefore, it has been postulated that during MCMV latency, low levels of viral gene expression/antigen presentation in latently infected cells, undergoing viral reactivation, would constantly stimulate inflationary CD8 T cells, which in turn would prematurely terminate such viral reactivation attempts [21], [30]. However, Snyder et al. showed that inflationary CD8 T cells divide only sporadically during latency and that they are not maintained in an antigen-dependent manner, thereby favoring a model where the inflationary pool is maintained by continuous replacement of newly activated effector CD8 T cells [29].

However, critical aspects regarding the mechanism of antigen presentation during MCMV latency are still unknown. We recently showed that contrary to the primary expansion during the acute phase of infection, which was largely driven by cross-presenting dendritic cells (DCs), accumulation and maintenance of the inflationary CD8 T cell pool occurred in absence of cross-presenting DCs, arguing for different antigen presentation requirements during memory inflation [31]. In addition, priming and inflation also differ in their costimulatory requirements, the latter being independent of B7-CD28 interaction [32]. Taking together, these data suggest that antigen presentation during latency might not depend on DCs, but on other cell types which are presumably hosting latent viral genomes and directly presenting viral antigens to the inflationary CD8 T cells.

A major obstacle in studying memory inflation is the lack of a monoclonal population of inflationary CD8 T cells that can be tracked *in vivo* during the entire course of MCMV infection. Therefore, we generated MHC class I-restricted TCR transgenic mouse lines with specificity for the MCMV-derived epitope M38, representing an immunodominant epitope of the inflationary response in C57BL/6 mice. Through a series of adoptive transfer experiments, we discovered that memory inflation was completely dependent on antigen presentation by non-hematopoietic cells. Consistent with this, we identified non-hematopoietic cells as major carriers of MCMV genomes during the whole course of the infection, especially during latency. While inflationary CD8 T cells with an effector phenotype accumulated during latency in the periphery without increased local proliferation, a small proportion of inflationary CD8 T cells in lymph nodes, displaying a T_CM_ phenotype, proliferated extensively during latency. We propose a mechanism where latently infected non-hematopoietic cells stochastically present viral antigens to T_CM_ CD8 T cells in lymph nodes, which differentiate into effector cells and migrate to peripheral tissues where they do not further proliferate but instead facilitate the control of local viral reactivation events.

## Results

### Tissue-specific pattern of MCMV-specific CD8 T cell responses

**Figure 1:**
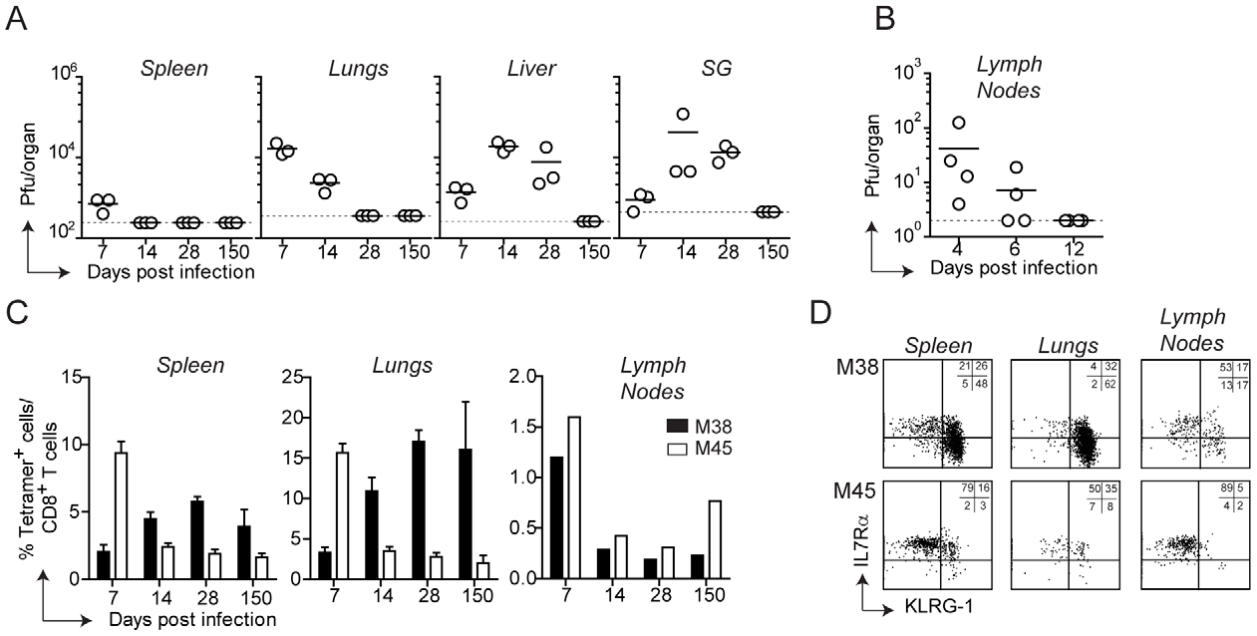
Tissue-specific pattern of MCMV-specific CD8 T cell responses. (A) C57BL/6 mice were infected with MCMV-Δm157 and virus titers were determined in the spleen, lungs, liver and salivary glands (SG) at days 7, 14, 28 and 150. In a separate experiment, virus titers were determined in the axillary lymph nodes at days 4, 6 and 12 post infection (B). The horizontal lines represent the respective detection limits. (C) The percentage of M38- (black bars) and M45-specific CD8 T cells (white bars) were measured by tetramer staining in the spleen, lungs and inguinal lymph nodes at the indicated time points post MCMV infection. For spleen and lungs, bars show averages of three mice per group ± SEM, whereas for lymph nodes bars represent cells pooled from three mice. (D) Representative plots showing KLRG-1 and IL7Rα expression on M38- and M45-specific CD8 T cells in the spleen, lungs and inguinal lymph nodes on day 150 post infection. All data are representative of at least three independent experiments.

Infection of C57BL/6 mice with MCMV results in viral replication which is controlled by the immune system with an organ-dependent kinetics: clearance of infectious virus was first observed in the spleen and in the lymph nodes, followed by the lungs, liver and lastly salivary glands, where lytic replication persisted for at least two months (Fig. 1A and B). By day 150 post infection infectious virus was no longer detected in any organ, despite the presence of latent viral genomes [33]. MCMV infection induces two distinct patterns of CD8 T cell responses: a conventional and an inflationary response. Conventional CD8 T cells, which are dominated by M45-specific CD8 T cells, contract after the resolution of the acute phase of infection to eventually generate low numbers of T_CM_ CD8 T cells (Fig. 1C). Inflationary CD8 T cells, exemplified by M38-specific CD8 T cells, accumulate until a stable plateau is reached. These two kinetic patterns were observed in the spleen and in peripheral organs such as lungs and liver (Fig. 1 C, liver not shown), where M45-specific CD8 T cells contracted between day 7 and day 14 post infection while M38-specific CD8 T cells increased until day 14–28 post infection to eventually stabilize at high percentages. In these organs, the majority of M45-specific CD8 T cells were IL7Rα^high^KLRG1^low^ and expressed high levels of CD62L (not shown) by day 150 after infection (Fig. 1D, bottom panels), reminiscent of a T_CM_ phenotype. In contrast, M38-specific CD8 T cells displayed largely an effector phenotype (IL7R^low^KLRG1^high^CD62L^low^), especially in the lungs (Fig 1 D, top panels) and in the liver (not shown). Interestingly, in the lymph nodes (here the inguinal LN is shown) M38-specific CD8 T cells did not inflate and were present at much lower percentages than in spleen and peripheral tissues (Fig. 1C). Moreover, about 50% of M38-specific CD8 T cells displayed a T_CM_ phenotype, independent of the anatomical location of the LNs analyzed (Fig. 1D and Fig. S1).

Thus, during MCMV infection two different CD8 T cell response patterns were induced, whose kinetics and phenotype were strongly influenced by their anatomical location. Strikingly, M38-specific CD8 T cells exhibited a pronounced T_EM_ phenotype in peripheral tissues and spleen whereas a predominant T_CM_ phenotype was observed in lymph nodes.

### Generation of MHC class I-restricted TCR transgenic mice with specificity for the M38 epitope of MCMV

To dissect the cellular and molecular requirements for memory inflation, adoptive transfer experiments of MCMV-specific CD8 T cell populations at various differentiation statuses and at various time points of infection are required, optimally using monoclonal T cell populations to normalize the heterogeneity inherent within total populations. Therefore, we generated TCR transgenic mouse lines with specificity for the MCMV-derived M38 epitope.

**Figure 2:**
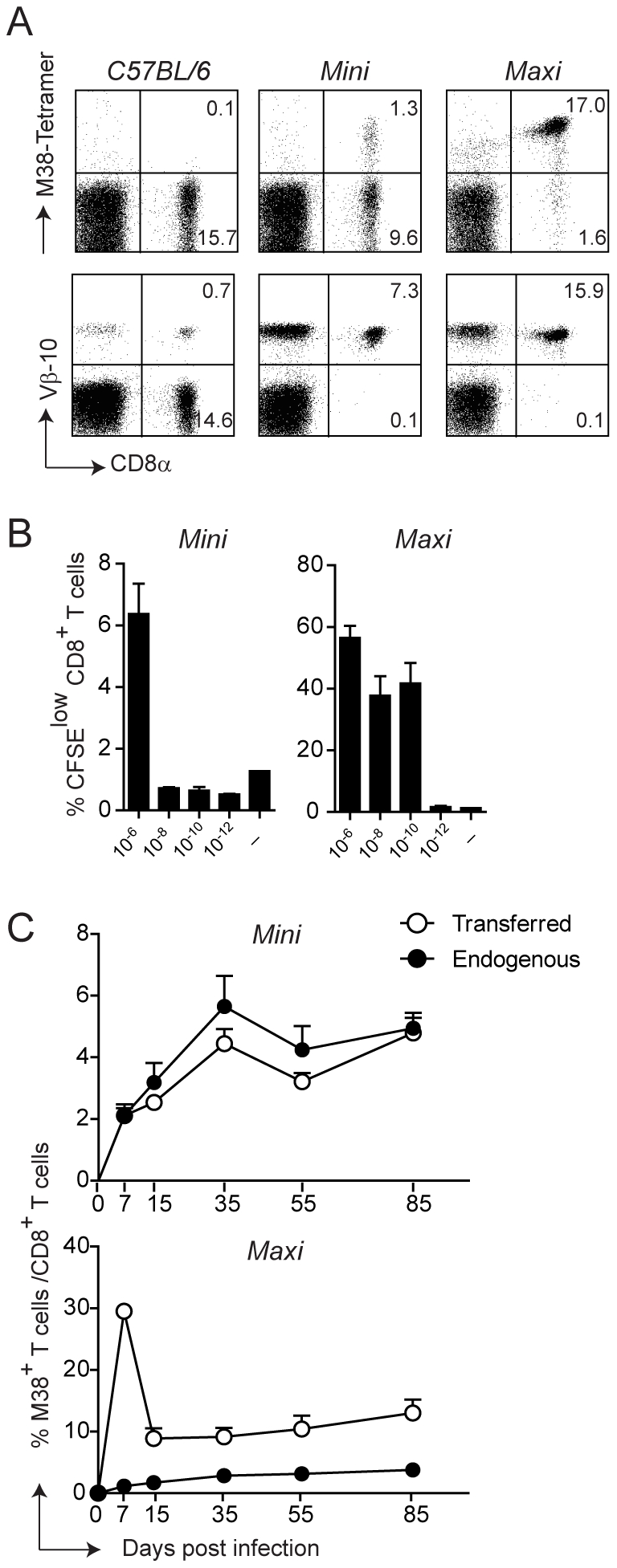
Generation of MHC class I-restricted TCR transgenic mice with specificity for the M38 epitope of MCMV. (A) Splenocytes from WT C57BL/6 mice and from the transgenic mouse lines Mini and Maxi were stained with M38-tetramer (top panels) and with anti-Vβ10 antibody (lower panels). Percentages of CD8^+^ M38^+^ and CD8^+^ Vβ10^+^ are depicted in the plots and are representative of at least 10 individual mice. (B) 6×10^5^ Mini CD8 T cells and 6×10^4^ Maxi CD8 T cells were CFSE labeled and incubated with DCs loaded with the indicated concentration of M38 peptide or with medium for three days. The percentages of CFSE^low^ cells are depicted. Bars show averages of three mice per group ± SEM, and one of two independent experiments is shown as representative. (C) 10^5^ Mini CD8 T cells or 10^4^ Maxi CD8 T cells expressing the CD45.1 congenic marker were adoptively transferred into CD45.2 recipient C57BL/6 mice one day prior to infection with MCMV. The percentages of endogenous (black circles) and transferred (white circles) M38-specific CD8 T cells were measured by tetramer staining from blood samples collected at the indicated time points after infection. Each point shows the average of three to five mice per group ± SEM. One of at least five (for Mini CD8 T cells) and two (for Maxi CD8 T cells) independent experiments are shown as representative.

For this, we first generated T cell hybridomas specific for the M38-derived epitope and focused on one high affinity hybridoma who’s TCR variable regions were identified as Vα4Jα13 and Vβ10Jβ2.1 (nomenclature according to Wilson et al., [34]). The rearranged genomic variable regions were amplified and cloned into TCR cosmid expression vectors which have been previously shown to allow efficient expression of transgenic TCRs [35]. Co-injection of the linearized transgenes into fertilized C57BL/6 oocytes gave rise to two different mouse lines, which we called Mini and Maxi (Fig. 2A). The Mini mouse was transgenic for the Vβ10Jβ2.1 chain only, which was expressed by nearly 100% of the CD8 T cells, while the TCRα chain was of endogenous origin (not shown). This increased the percentage of naïve M38-specific CD8 T cells from undetectable levels in C57BL/6 mice to ∼10%. The Maxi mouse was transgenic for both Vα4Jα13 and Vβ10Jβ2.1 variable regions, resulting in a percentage of M38-specific CD8 T cells among total naïve CD8 T cells of around 90% (Fig. 2A).

To compare the relative affinities of the TCRs expressed by the Mini and Maxi CD8 T cells, we first analyzed their dose response to graded amounts of M38 peptide *in vitro*. Both Mini and Maxi CD8 T cells were specific for the M38 peptide, though Maxi CD8 T cells had a 10′000-fold higher affinity compared to Mini CD8 T cells as identified by *in vitro* proliferation (Fig. 2B). To determine the ability of transgenic CD8 T cells to proliferate *in vivo* in response to MCMV infection, we adoptively transferred 10^5^ Mini or 10^4^ Maxi CD8 T cells into C57BL/6 mice one day prior to infection and compared endogenous and transgenic M38-specific CD8 T cell responses in the blood at different time points after infection (Fig. 2C). Adoptively transferred cells were distinguished from endogenous cells by expression of the CD45.1 congenic marker. Mini CD8 T cells responded to MCMV infection comparable to the endogenous M38-specific CD8 T cells over the whole course of the infection, showing inflation and maintenance at high percentages during latency with a T_EM_ phenotype (Fig. S2). The much higher affinity of the TCR expressed by Maxi CD8 T cells resulted in a 10-fold higher expansion during the acute phase of infection compared to equivalent starting numbers of transferred Mini CD8 T cells. Interestingly, the classical inflationary behavior was not observed for the Maxi CD8 T cells, as a contraction phase followed the expansion phase. Nevertheless, Maxi CD8 T cells were still maintained at high percentages during latency and displayed a T_EM_ phenotype, indicative of the inflationary response (Fig. S2).

Thus, the Maxi mouse is a source of a monoclonal population of CD8 T cells that expresses a TCR with an exquisitely high affinity for the M38-derived antigen. In contrast, CD8 T cells that originate in the Mini mouse are highly enriched in M38-specific CD8 T cells and are rather polyclonal, which renders them well suited for adoptive transfer experiments mimicking the endogenous response that is induced *in vivo* upon MCMV infection.

### Antigen presentation by non-hematopoietic cells is essential for M38-specific CD8 T cell inflation

**Figure 3:**
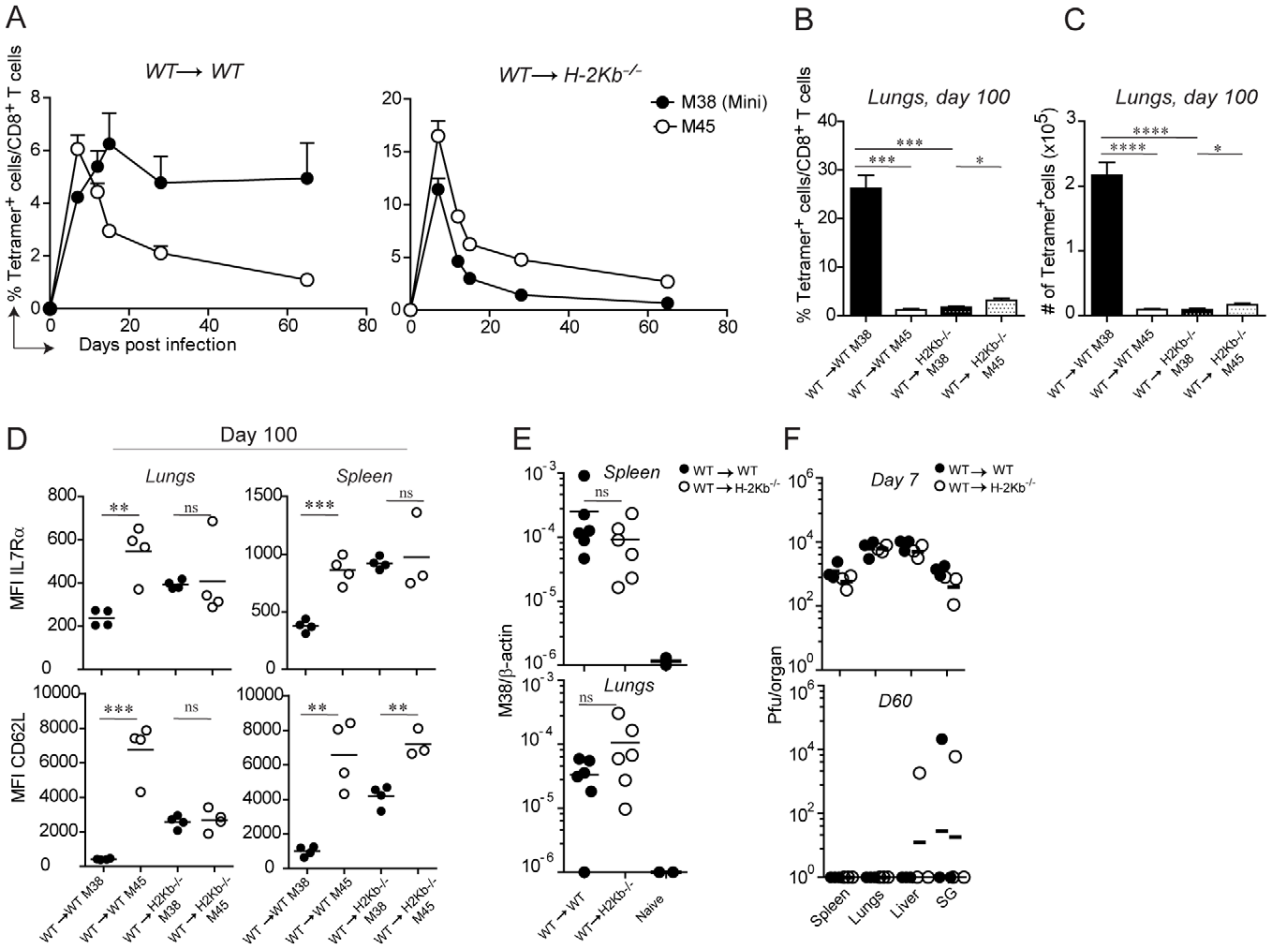
Antigen presentation by non-hematopoietic cells is essential for M38-specific CD8 T cell inflation. WT→WT and WT→H-2K^b−/−^ chimeric mice were generated by irradiating WT and H-2K^b−/−^ mice followed by reconstitution with WT bone-marrow. One day prior infection with MCMV-Δm157, 10^5^ CD45.1^+^ Mini CD8 T cells were adoptively transferred. (A) The percentage of M38- (pregated on CD45.1^+^ cells) and M45-specific CD8 T cells among total CD8 T cells were measured in the blood on days 7, 12, 15, 28 and 65 post infection. Every point represents the average of four individual mice ± SEM. The mice shown in (A) were sacrificed on day 100 post infection and the percentage of M38- (pregated on CD45.1^+^ cells) and M45-specific CD8 T cells among total CD8 T cells (B) and their total numbers (C) were determined in the lungs. Bars indicate averages of four mice per group ± SEM. The relative expression of IL7Rα and CD62L on M38- and M45- specific CD8 T cells is indicated in (D) in the same groups of mice shown in (A) for lungs and spleen. (E) MCMV latent genomes were quantified in the spleen (upper panel) and in the lungs (lower panel) of WT→WT (black circles) and WT→H-2K^b−/−^ mice (white circles) on day 60 post infection or from naïve mice by qPCRs using primers specific for β-actin and the MCMV-encoded M38 gene. The relative amount of viral genome was calculated using the delta-delta Ct method, where 10^−6^ was the highest value detected for naïve mice and used as detection limit. Data pooled from two independent experiments are shown. (F) Virus titers were determined in spleen, lungs, liver and salivary glands on day 7 (top panel) and day 60 (bottom panel) in WT→WT (black circles) and WT→H-2K^b−/−^ (white circles) mice. Lines represent the geometric means of three individual mice. One of at least four independent experiments are shown for (A to D) and of two independent experiments for (E to F). Significances were analyzed by Student's t test. *, P<0.05; **, P<0.01; *** P<0.001; ****, P<0.0001.

With the assumption that memory inflation is dependent on MHC class I-antigen complex recognition and with the availability of the TCR transgenic CD8 T cells, we sought to determine which cell types are responsible for antigen presentation to inflationary CD8 T cells. In particular, we addressed the question whether H-2K^b^ restricted M38-specific CD8 T cells require antigen presentation by hematopoietic or non-hematopoietic cells for their inflation. To this end, we generated bone marrow chimeras in which the MHC class I H-2K^b^ molecule was expressed on every cell type (WT→WT) or selectively removed on non-hematopoietic cells (WT→H-2K^b−/−^), and compared these two groups of mice for their kinetics of M38- and M45-specific (H-2D^b^-restricted) CD8 T cell responses. To overcome the absence of positive selection of H-2K^b^ restricted CD8 T cells in H-2K^b−/−^ recipient mice, we adoptively transferred congenically marked Mini CD8 T cells the day prior to infection. We first compared the kinetics of M38- and M45-specific CD8 T cell responses in the blood (Fig. 3A). As expected, in WT→WT mice M45-specific CD8 T cells underwent the classical expansion-contraction kinetics typical of a conventional response, while M38-specific CD8 T cells expanded until day 15 post infection, after which they stabilized at high percentages (Fig. 3A, left graph). Strikingly, memory inflation was not at all induced in WT→H-2K^b−/−^ mice (Fig. 3A, right graph) in the absence of antigen presentation by non-hematopoietic cells. Instead, M38-specific CD8 T cells underwent classical expansion and contraction kinetics as observed for M45-specific CD8 T cells.

Next, we investigated whether antigen presentation by non-hematopoietic cells was also a prerequisite for memory inflation in peripheral tissues. Consistent with the blood data, we measured a 20-fold reduction of M38-specific CD8 T cells in WT→H-2K^b−/−^ compared to WT→WT mice (Fig. 3B and C) in the lungs of latently infected mice. Similar results were obtained in the spleen and in the liver (not shown). A hallmark of inflationary CD8 T cells is their activated phenotype, which has been suggested to be a consequence of presentation of viral-derived antigens during latency. If this is true, then absence of antigen sensing on non-hematopoietic cells would favor the formation of T_CM_ M38-specific CD8 T cells during latency. This was indeed the case: in both the spleen and the lungs of latently infected WT→ H-2K^b−/−^ mice, M38-specific CD8 T cells up-regulated the expression of IL7Rα and CD62L to a similar extent as in M45-specific CD8 T cells. M38-specific CD8 T cells also displayed high expression of CD122 in WT→H-2K^b−/−^ mice, allowing for IL-15-mediated homeostatic proliferation (not shown). Importantly, this was the case despite comparable abundance of viral genomes in the spleen and lungs of WT→WT and WT→H-2K^b−/−^ mice on day 60 post infection (Fig. 3E), indicating that latency was established in WT→H-2K^b−/−^ mice and that absence of memory inflation was due to impaired/missing antigen presentation. Moreover, virus titers were comparable on day 7 post infection, and by day 60 post infection virus control was almost complete in every organ analyzed in both WT→WT and WT→H-2K^b−/−^ chimeras (Fig. 3F).

These data show that antigen presentation by non-hematopoietic cells is a prerequisite for memory inflation during MCMV infection. In its absence, inflationary CD8 T cells are undistinguishable from the conventional CD8 T cells with respect to their kinetics and phenotypes.

### Restriction of antigen presentation to DCs abrogates memory inflation

**Figure 4:**
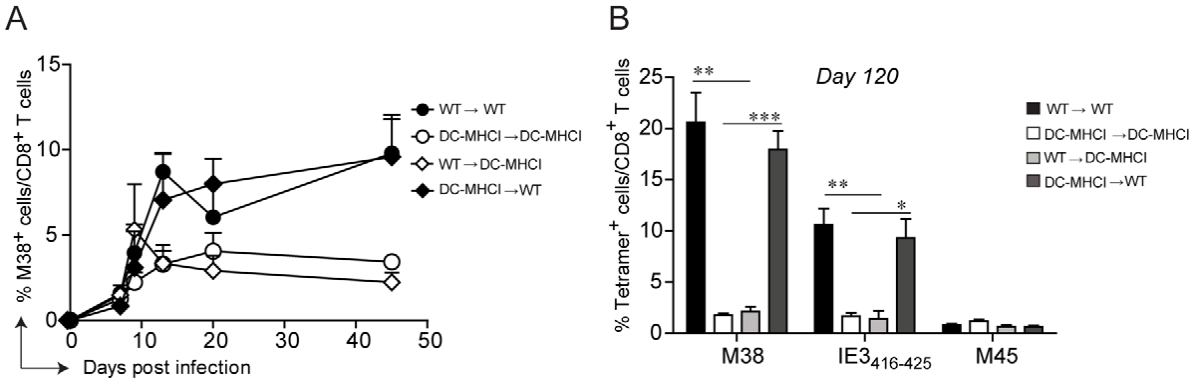
Restriction of antigen presentation to DCs abrogates memory inflation. WT→WT, DC-MHCI→DC-MHCI, WT→DC-MHCI, DC-MHCI→WT chimeric mice were generated by reconstituting WT and DC-MHCI irradiated mice with WT and DC-MHCI bone marrow. All groups (3–5 mice per group) were infected with MCMV-Δm157 and the M38-specific CD8 T cell response was measured by tetramer staining from blood samples obtained on days 7, 9, 13, 20 and 45 post infection (A). The experiment was terminated on day 120 post infection and M38-, IE3-, and M45-specific CD8 T cell responses were quantified in the lungs by tetramer staining (B). One of two independent experiments is shown. Significances were analyzed by Student's t test. *, P<0.05; **, P<0.01; *** P<0.001.

Memory inflation is not solely restricted to CD8 T cells specific for the M38-derived antigen. In C57BL/6 mice, at least three other MCMV-derived antigens have been identified that induce CD8 T cell inflation, namely IE3_416–425_, IE3_461–475_, and m139. To investigate whether antigen presentation by non-hematopoietic cells is a general requirement for memory inflation, we took advantage of *Tg(CD11c-β_2_m)*×*Tg(K14-β_2_m)*×*β_2_m* ^−*/*−^ mice (DC-MHCI) [36], [37]. These mice are β_2_-microglobulin^−/−^ mice with a tissue specific expression of transgenic β2-microglobulin in dendritic cells (DCs), on keratinocytes and on the thymic cortical epithelium, ensuring positive selection of CD8 T cells in the thymus. As a result, only DCs (and keratinocytes) are able to present antigens in secondary lymphoid organs and peripheral tissues in the context of MHC class I molecules in DC-MHCI mice. The presence of a normal peripheral CD8 T cell compartment in these mice [36] allows the assessment of endogenous H-2K^b^ and H-2D^b^-restricted CD8 T cell responses. Similarly as described above, WT and DC-MHCI mice were lethally irradiated and reconstituted with bone-marrow derived from WT mice (WT→WT and WT→ DC-MHCI) or from DC-MHCI mice (DC-MHCI →WT and DC-MHCI → DC-MHCI). We first analyzed the M38-specific CD8 T cell response in the blood, which confirmed the previous results and verified the validity of this model (Fig. 4A). In fact, memory inflation was only induced in the two groups of mice where the recipients were WT, irrespective of the origin of the hematopoietic cells, but not in DC-MHCI recipient mice. Notably, when DCs were the only cell type capable of presenting viral antigens (DC-MHCI → DC-MHCI), this had no effect on the expansion of M45-specific CD8 T cells during acute infection and showed only a minor reduction for M38-specific CD8 T cells (Fig. S3), implying that DCs alone are sufficient for priming and expansion of MCMV-specific CD8 T cell responses, but not for their inflation. To investigate the consequences of selective depletion of antigen presentation by non-hematopoietic cells for inflation of CD8 T cells specific for other antigens, we measured the responses to M38 and to IE3_416–425_ in the lungs of mice which had been infected for 120 days (Fig. 4B). We found a similar pattern of responses for the two inflators, namely a 5–10-fold reduction in DC-MHCI compared to WT recipient mice, suggesting that inflation of IE3-specific CD8 T cells was also dependent on antigen presentation by non-hematopoietic cells. Consistent with the data shown above, absence of antigen-presentation by non-hematopoietic cells resulted in up-regulation of IL-7Rα on M38- and IE3-specific CD8 T cells during latency (not shown). Thus, DCs are sufficient for priming of both the conventional and inflationary response, whereas inflation requires antigen presentation by cells of non-hematopoietic origin.

### Non-hematopoietic cells promote extensive and systemic cell proliferation in the early phase of MCMV infection, but only in lymph nodes during latency

**Figure 5:**
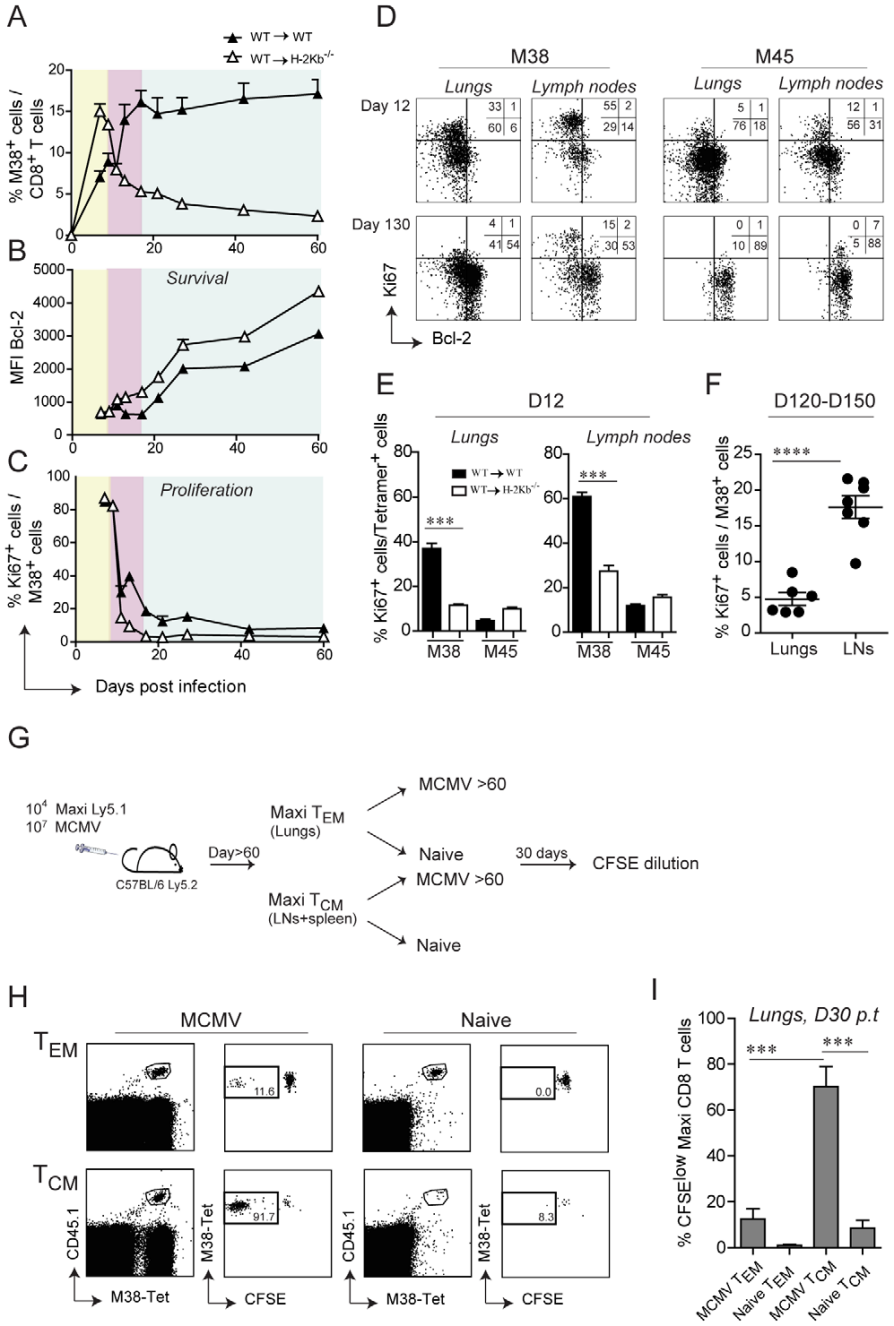
Non-hematopoietic cells promote extensive and systemic cell proliferation in the early phase of MCMV infection, but only local during latency. 10^5^ Mini-CD8 T cells were transferred in WT→WT and WT→H-2K^b−/−^ chimeric mice one day prior to infection with MCMV-Δm157. (A) The percentage of M38-specific CD8 T cell response was measured in the blood on days 7, 9, 11, 13, 17, 21, 27, 42 and 60 post infection. Colors indicate different phases of the M38-specific response: primary expansion in yellow; secondary expansion in pink; maintenance phase in blue. Bcl-2 (B) and Ki67 (C) expression levels were measured on M38-specific CD8 T cells depicted in (A). One of two independent experiments is shown. (D) Representative FACS plots showing Bcl-2 and Ki67 expression on M38- and M45-specific CD8 T cells in the lungs and inguinal lymph nodes of WT→WT chimeric mice on day 12 post infection (upper panels) and day 130 post infection (lower panels). (E) The percentage of ki67^+^ cells in WT→WT and WT→H-2K^b−/−^ chimeric mice is shown for M38- and M45-specific CD8 T cells in the lungs (left graph) and in the lymph nodes (right graph). One of three independent experiments is shown. (F) The percentage of M38-specific cells expressing Ki67 during latency is shown for the lungs and the lymph nodes. Data pooled from three independent experiments ± SEM are shown. (G) Preferential antigen-driven proliferation of T_CM_ M38-specific CD8 T cells during latency. Experimental layout: CD45.2^+^ C57BL/6 mice were adoptively transferred with CD45.1^+^ Maxi CD8 T cells and during latency T_CM_ Maxi CD8 T were isolated from the LNs and the spleen, whereas T_EM_ Maxi CD8 T cells were isolated from the lungs. Isolated cells were labeled with CFSE and transferred into naïve or latently infected CD45.2^+^ C57BL/6 mice (Number of transferred cells: T_CM_ = 20′000/mouse, T_EM_ = 80′000/mouse). CFSE dilution was measured 30 days later in the lungs. (H) Representative plots showing the population of transferred cells for each of the four experimental groups (gated on total CD8 T cell) and their respective CFSE dilutions, and (I) Graphical summary showing percentage of CFSE^low^ cells among total transferred Maxi CD8 T cells. Data in (I) are pooled from two independent experiments.

By careful kinetic analysis, the inflationary M38-specific CD8 T cell response can be divided into three consecutive phases: the primary expansion between day 0 and 9 post infection (Fig. 5A, yellow area), a first and most pronounced inflation (secondary expansion) from day 11 and day 15 post infection (Fig. 5A, pink area) followed by a maintenance phase (Fig, 5A, blue area) (Fig. 5A). In contrast, in WT→H-2K^b−/−^ mice primary expansion was followed by contraction instead of secondary expansion, suggesting that antigen presentation by non-hematopoietic cells promotes either survival of the effector cells or/and prolongs cell proliferation. To distinguish between these two possibilities, we first analyzed the expression profiles of the anti-apoptotic molecule Bcl-2 in adoptively transferred Mini CD8 T cells in WT→WT and WT→H-2Kb^−/−^ mice over the course of MCMV infection. At the peak of primary expansion, Mini CD8 T cells down-regulated Bcl-2 expression in both groups of mice, consistent with their highly activated phenotype (Fig. 5B, yellow area). However, contraction of Mini CD8 T cells in WT→H-2K^b−/−^ mice resulted in a continuous up-regulation of Bcl-2, comparable to conventional M45-specific CD8 T cells, whereas Mini CD8 T cells remained Bcl-2^low^ in WT→WT mice during secondary expansion (Fig. 5B, pink area and Fig. S4). These data are in line with the notion that absence of antigen presentation by non-hematopoietic cells abrogates inflation and results in generation of long-lived M38-specific T_CM_ CD8 T cells during latency. Unexpectedly, even in WT→WT mice where contraction never happens, Mini CD8 T cells started to up-regulate the expression of Bcl-2 at the onset of the maintenance phase, despite their phenotype remaining activated (Fig. 5B, blue area). Likewise, we observed a constant up-regulation of Bcl-2 expression by the endogenous M38-specific CD8 T cells (and as expected by M45-specific CD8 T cells) in the lungs (Fig. S4). From these data we exclude that non-hematopoietic cells provide surviving stimuli to inflationary CD8 T cells at the peak of the primary expansion preventing their contraction, instead they might deliver additional antigen stimulation resulting in prolonged cell activation and proliferation. This was confirmed by the expression profile of the proliferative marker Ki67 which was concomitantly analyzed with Bcl-2 expression. Indeed, Mini CD8 T cells underwent a second round of proliferation in WT→WT mice exactly at the time when secondary expansion was induced (Fig. 5C, pink area), but not in WT→H-2K^b−/−^ mice, where Ki67^+^ Mini cells sharply decreased during the contraction phase and remained low for the rest of the infection. Consistent with the results by Snyder et al. who showed that inflationary CD8 T cells were dividing only sporadically during latency [29], we observed a decrease of proliferating Mini CD8 T cells at the onset of the maintenance phase in WT→WT mice (Fig. 5C, blue area) to eventually reach the low level of proliferation measured in WT→H-2K^b−/−^ as latency was established (D60: WT→WT: 7%; WT→H-2K^b−/−^: 3%).

Next, we analyzed whether the proliferation pattern observed in the blood was also apparent in lymphoid and peripheral tissues. We therefore analyzed Ki67/Bcl-2 expression in M38-specific and M45-specific CD8 T cells isolated from lungs or lymph nodes on day 12 post infection and during latency (Fig. 5D, E and F). Analogous to the blood, M38-specific CD8 T cells in WT→WT mice showed increased proliferation compared to WT→H-2K^b−/−^ mice on day 12 post infection, especially in the lymph nodes (Fig. 5E). Strikingly, by 4 months post infection (Fig. 5D and F) M38-specific cell proliferation had ceased almost completely in the lungs as was the case for M45-specific cells, while it was still ongoing at high levels in the lymph nodes. Importantly, while the few Ki67^+^ M45-specific CD8 T cells in the lymph nodes were Bcl-2^high^, indicative of cytokine-driven homeostatic proliferation, proliferating M38-specific CD8 T cells were Bcl-2^low^, suggesting that their proliferation was antigen driven. Since M38-specific CD8 T cells display largely a T_CM_ phenotype in lymph nodes, our data strongly suggest that central-memory cells in the lymph node, and not effector memory cells in peripheral tissues, respond to antigen presentation on non-hematopoietic cells during latency by proliferation. To test this hypothesis, we adoptively transferred *in vivo* generated T_EM_ or T_CM_ M38-specific CD8 T cells into latently infected mice or naïve mice, and assessed their proliferative history 30 days later (Fig. 5G). As expected, T_CM_ M38-specific CD8 T cells proliferated to a much higher extent compared to T_EM_ M38-specific CD8 T cells in latently infected recipients (Fig. 5H and I).

Together, these data suggest that antigen presentation by non-hematopoietic cells promote extensive and systemic proliferation of M38-specific CD8 T cells during the early phase of the infection, resulting in their secondary expansion. However, subsequent maintenance of the peripheral inflationary pool at high percentage during latency cannot be attributed to extensive systemic cell proliferation, but instead might be facilitated by prolonged survival of the inflationary cells, perhaps facilitated by Bcl-2 upregulation, and by continuous recruitment of newly activated central memory CD8 T cells from lymph nodes.

### MCMV genomes preferentially localize in non-hematopoietic cells

**Figure 6:**
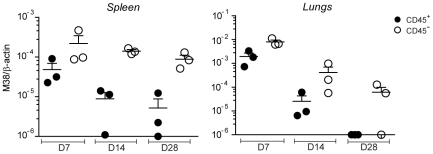
MCMV genome localizes preferentially in non-hematopoietic cells. C57BL/6 mice were infected with MCMV-Δm157 and on day 7, 14 and 28 post infection total cells from the spleen and the lungs were sorted into CD45^+^ (black circles) and CD45^-^ (white circles) cell populations. MCMV latent genomes were quantified by qPCR using primers specific for β-actin and the MCMV-encoded M38 gene. One of two independent experiments is shown.

Given that priming and memory inflation rely on antigen presentation by different cell types, the first being dependent on DCs and the latter on non-hematopoietic cells, it is conceivable that productive infection during the acute phase of infection and establishment of latency occurred in different cell types. We aimed to analyze the target cells of MCMV distinguishing between hematopoietic cells or non-hematopoietic cells in secondary lymphoid organs and in peripheral tissues at different time points of the infection. To this end, we isolated spleen and lungs from WT mice on day 7, 14 and 28 after infection and stained the isolated cells with anti-CD45 antibody, a known marker for hematopoietic cells. The proportion of cells that stained positive for CD45 was roughly ∼80% in the spleen and ∼60% in the lungs (not shown). We then sorted CD45-positive and CD45-negative cell populations and measured the relative MCMV genome content by quantitative real time PCR (qPCR). In both the spleen and the lungs, we found a clear bias towards the CD45-negative population at every time point analyzed (Fig 6). Similar results were observed in LNs, when sufficient DNA was recovered from non-hematopoietic cells for accurate qPCR amplification (data not shown). While MCMV genomes were also found in the CD45-positive population on day 7 and 14 post infection, when productive infection was still ongoing, they were hardly detectable on day 28, a time point when the virus had already established latency in both the spleen and the lungs (Fig. 1A). Thus, during the acute phase of the infection, when CD8 T cells of both the conventional and inflationary type are primed, MCMV genomes are found in both hematopoietic and non-hematopoietic cell compartments. At later time points, when the inflationary memory CD8 T cell response is induced, MCMV localizes predominantly in non-hematopoietic cells, strengthening the hypothesis that latently infected non-hematopoietic cells directly present viral-derived antigens during latency, leading to memory inflation.

## Discussion

MCMV infection induces accumulation and life-long peripheral maintenance of a population of virus-specific CD8 T cells with an activated phenotype, a phenomenon referred to as ‘memory inflation’ [25]. A major population of these inflationary CD8 T cells in the C57BL/6 background is specific for the MCMV-derived epitope M38 [28]. The cellular and molecular requirements leading to memory inflation are still poorly understood, mainly due to lack of monoclonal populations of MCMV-specific CD8 T cells that can be traced *in vivo* during the course of the infection. In the present study we describe the generation of two MHC class I-restricted mouse lines with specificities for the M38-epitope of MCMV, which differ in their affinity towards the peptide. Through a series of adoptive transfer experiments we could demonstrate that i) memory inflation is completely dependent on antigen presentation by cells of non-hematopoietic origin, which was recently also reported for mice of non-C57BL/6 background [38], ii) only in lymph nodes inflationary CD8 T cells respond to continued antigen presentation by proliferation whereas antigen presentation by non-hematopoietic cells in peripheral tissues might contribute to their long term effector phenotype in these tissues during latency, and iii) non-hematopoietic cells are the major reservoir of MCMV latent genomes after resolution of acute infection. We propose a model where memory inflation is induced and maintained by latently infected non-hematopoietic cells that directly restimulate inflationary CD8 T cells with a T_CM_ phenotype in lymph nodes, inducing secondary expansion and migration in the periphery.

This model also provides an attractive explanation to why two patterns of CD8 T cell responses are induced during MCMV infection, if combined with a recent finding by Hutchinson et al, who showed that conventional CD8 T cells displayed a higher dependence on the immunoproteasome subunit LMP7 compared to inflationary CD8 T cells [39]. The immunoproteaseome is constitutively expressed by immune cells like DCs and macrophages, whereas expression in non-immune cells is only induced by IFNγ [40]. Thus, in non-inflammatory conditions as during MCMV latency, infected non-hematopoietic cells would only process proteins through the conventional immunoproteasome.

Inflationary CD8 T cells, in contrast to conventional CD8 T cells, do not contract upon control of lytic MCMV replication but rather continuously accumulate even in the absence of overt viral replication [27], [28]. Systematic longitudinal proliferation analyses allowed the subdivision of the M38-specific CD8 T cell response into three consecutive phases: a primary expansion characterized by extensive cell proliferation, immediately followed by a secondary expansion with reduced but substantial proliferation, and a maintenance phase, where M38-specific CD8 T cells stabilized in number and underwent very little proliferation - with the notable exception of lymph nodes. Primary expansion of M38-specific CD8 T cells occurred in response to acute infection and showed high similarities to the conventional M45-specific CD8 T cell response. In fact, both responses almost completely relied on antigen presentation by DCs and generated effector cells with similar effector functions and activation state [28], [41]. The two responses diverged at the peak of the primary expansion, as conventional CD8 T cells contracted and inflationary CD8 T cells underwent secondary expansion. Strikingly, secondary expansion was promoted by robust proliferation which was entirely dependent on non-hematopoietic cells. Two possible mechanisms could account for the ability of non-hematopoietic cells to induce proliferation of inflationary CD8 T cells: one is that infected non-hematopoietic cells in the periphery perpetuate clonal expansion that is initiated in the secondary lymphoid organs, as described for acute LCMV infection [42]. The second is that secondary expansion is induced in lymph nodes by infected non-hematopoietic cells, re-stimulating inflationary CD8 T cells with a T_CM_ phenotype, thereby inducing reactivation and migration to the periphery. Our data support the second hypothesis as the highest proliferation levels during secondary expansion were not detected in peripheral organs such as lungs or liver where massive accumulation of inflationary CD8 T cells was observed, but in the lymph nodes, where M38-specific CD8 T cells did not inflate and possessed largely a T_CM_ phenotype. However, since the two hypotheses are not mutually exclusive, we do not formally rule out that a certain degree of proliferation might also be induced in peripheral tissues. Furthermore, although our data clearly show a strict dependence of antigen recognition on non-hematopoietic cells for memory inflation to occur, it is possible that memory inflation could also be stimulated by hematopoietic cells, albeit at undetectable levels because there might not be enough latently infected hematopoietic cells to cause inflation on their own.

The proposed scenario where non-hematopoietic cells directly restimulate T_CM_ CD8 T cells in lymph nodes and induce their secondary expansion seems reasonable, as it is well-known that memory CD8 T cells are less stringent in terms of costimulatory requirements compared to their naïve counterparts and that T_CM_ CD8 T cells are by far superior in proliferative recall responses as opposed to T_EM_ CD8 T cells [6], [43], [44]. Moreover, we recently showed that memory inflation, in contrast to priming, was largely independent of CD8α^+^ and CD103^+^ DCs [31], which are the major subsets of cross-presenting DCs described so far, suggesting that memory inflation is largely driven by direct antigen presentation. Last but not least, non-hematopoietic cells are the major reservoir of MCMV latent genomes after resolution of acute infection in both peripheral and secondary lymphoid organs [45], [46].

While it seems evident that secondary expansion is antigen-dependent and characterized by extensive proliferation, it is still unclear what the requirements are for maintenance of the inflationary pool in the periphery once that true latency is established. By five months post infection, proliferation of inflationary CD8 T cells is almost undetectable in the lungs, although they are maintained at high numbers in this peripheral tissue. A number of recent reports are consolidating the notion that so called tissue-resident memory T cells with an effector memory phenotype can persist in the periphery for long time periods in the absence of the cognate antigen [7], [8], [47], [48]. Whether this also applies for MCMV infection remains to be shown. However, long-term maintenance of virus-specific CD8 T cells are likely to play an important role during latent infections, where they would provide a key protective role in active immune surveillance controlling virus reactivation events, as demonstrated for HSV infection [8]. The cellular requirements ensuring survival of these effector cells are still undefined; CD103 expression on CD8 T cells seems to play a critical role for HSV and other infections [7], [8], but probably not during MCMV as inflationary CD8 T cells do not express CD103 (data not shown). However, inflationary CD8 T cells upregulate Bcl-2, also in peripheral tissues and despite exhibiting a T_EM_ phenotype, suggesting that cytokines or other stimuli might promote their survival. Yet, it is unlikely that the inflationary pool is maintained lifelong without being replenished, as already suggested by Snyder et al [29]. In line with this, we found a high percentage of proliferating M38-specific CD8 T cells in the lymph nodes during latency. Moreover, T_CM_ M38-specific CD8 T cells as opposed to T_EM_ CD8 T cells proliferated in an antigen-dependent manner when transferred into a latently infected host. Together, these data indicate that re-stimulation of T_CM_ memory cells by non-hematopoietic cells in the lymph nodes might continuously supply the inflationary pool in the periphery with new and functional effector cells.

## Materials and Methods

### Ethics statement

This study was carried out in in strict accordance to the guidelines of the animal experimentation law (SR 455.163; TVV) of the Swiss Federal Government. The protocol was approved by Cantonal Veterinary Office of the canton of Zurich, Switzerland (Permit number 145/2008). All surgery was performed under isoflurane anesthesia and all efforts were made to minimize suffering.

### Mice, viruses and immunization

C57BL/6N, H-2K^b−/−^ and *Tg(CD11c-β_2_m) x Tg(K14-β_2_m) x β_2_m* ^−*/*−^ (DC-MHCI) mice were bred in the local animal facility under specific pathogen-free conditions. KM14 mice were kindly provided by Prof. T. Brocker (Ludwig-Maximilians-University, Munich). Recombinant MCMV-Δm157 (*m157* deletion mutant) was described previously [49] and was grown on C57BL/6 embryonic fibroblasts (MEFs) and titrated by standard plaque-forming assays as described in [50]. Infection was performed intravenously (i.v.) with 10^7^ plaque forming units (PFU) of MCMV-Δm157.

### Generation of Mini and Maxi transgenic mice

#### A) Generation of M38_316–323_-specific CD8 T cell hybridomas

C57BL/6N mice were infected intravenously with MCMV-Δm157 and at least 60 days later spleen cells were isolated and activated for 8 days with 10^−8^ M of M38_316–323_ peptide (NeoMPS, Strasbourg, France) in presence of 80U/ml of recombinant IL-2 (BD Biosciences, Basel, Switzerland). Another restimulation followed, and three days later cells were fused with BW2 36.1 CD8α cell line (kindly provided by Prof. Marcus Groettrup, University of Konstanz, Germany) according to the protocol described in [51]. Specificity of the CD8 T cell hybridomas was assessed as described in [49]. The hybridoma with the highest peptide affinity was selected for further characterization.

#### b) Identification of TCRVα and TCRVβ variable region genes and generation of TCR transgenic mouse lines

RNA was isolated form the selected hybridoma using TRIzol reagent (Invitrogen, Basel, Switzerland) according to the manufacturer's instructions, and cDNA was generated using M-MLV Reverse Transcriptase RNase, H Minus (Promega, Dübendorf, Switzerland). cDNA was amplified with a TCRα-specific primer set [52] and a TCRβ-specific primer set [53]. Sequencing of the PCR products was done by Microsynth (Zürich, Switzerland) and then aligned to the mouse genome using Ensemble database (http://www.ensembl.org/Mus_musculus). The identified Vα4Jα13 and Vβ10Jβ2.1 gene segments were amplified from the genomic DNA using the following primers: for Vα4Jα13: fwd (5′-TGA CCC GGG TTC TAG ATG ACA CTA AAG ATG G-3′), TCRα rev (5′-ATA TGC GGC CGC ACA ATT CAG ACA TGG ACT TAC-3′), for Vβ10Jβ2.1: fwd (5′-TAA CTC GAG GCT TAT TTG CCC TGC CTT GAC CCA ACT ATG-3′), rev (5′-TTT CCG CGG CTC CCA CCT GTA TGG CCT CTG CCT TCT TAC CTA-3′). PCR products containing Vα4Jα13 and Vβ10Jβ2.1 gene segments were digested with XmaI and NotI respectively with XhoI and SacII and cloned into previously described TCR expression vectors [35]. The resulting pTαVα4Jα13 and pTβVβ10Jβ2.1 were digested with SalI respectively KpnI to excise the transgenes from prokaryotic vector DNA. The isolated linearized fragments were co-injected in equimolar ratios into fertilized C57BL/6N oocytes according to the standard method [54]. The resulting two TCR transgenic mouse lines were designated according to the standardized genetic nomenclature for mice: C57BL/6N-*Tg(Tcrb)330Biat* (Mini) and C57BL/6N-*Tg(Tcra,Tcrb)329Biat* (Maxi) [55].

### Generation of bone-marrow chimeras

Chimeric mice were generated by transferring 2–5×10^6^ bone marrow cells derived from C57BL/6N or DC-MHCI mice 6 to 8 hours after lethal irradiation (950 rad) of recipient mice (C57BL/6N, H-2K^b−/−^ or DC-MHCI mice). During the first two weeks of reconstitution, mice were treated with the antibacterial Borgal 24% (Intervet, Boxmeer, Netherlands). Bone marrow chimeric mice were used for experiments 8–10 weeks after reconstitution.

### Antibodies and tetramers

APC-conjugated peptide-MHC class I tetramers were generated as described in [56]. The following conjugated antibodies were purchased either from Biolegend (Lucerna Chem AG, Luzern, Switzerland) or from BD Biosciences (Allschwil, Switzerland): anti-CD8α [53−6.7], anti-CD127 [SB/199], anti-CD62L [MEL-14], anti-KLRG-1 [2F1], anti-Vβ10 [B21.5], anti-CD45.1 [A20], anti-CD45.2 [104], anti-CD44 [IM7], anti-Ki67 [B56], anti-Bcl-2 [3F11].

### Lymphocyte isolation, cell surface and intracellular staining

Lymphocytes were isolated from spleen, lymph nodes, lungs and liver as described in [57]. Fresh blood was obtained from the tail vein. For surface staining, cells were incubated for 20 min at 4°C or at 37°C when using tetramers before lysis with 1X BD lysis buffer (BD Biosciences) for 5 min at room temperature. For intracellular staining of Ki67 and Bcl-2, cells were first permeabilized by incubation in 1X wash/perm buffer (BD Biosciences) for 15 minutes at 4°C. Staining was then performed in 1X wash/perm buffer containing the appropriate antibody dilutions for 30 min at 4°C. Multiparameter flow cytometric analysis was performed using a LSRII flow cytometer (BD Biosciences) and analyzed with FlowJo software (Tree Star, San Carlos, CA).

### Adoptive transfer experiments

CD8 T cells were purified from Mini- or Maxi- derived splenocytes with anti-CD8α MACS beads (Miltenyi Biotech, Bergisch Gladbach, Germany) according to the manufacturer's instructions and 10^5^ Mini- or 10^4^ Maxi-CD8 T cells were adoptively transferred into recipient mice one day prior to infection. For transfer of different CD8 T cell memory subsets, 10^4^ MAXI CD8 T cells were first adoptively transferred into recipient mice one day prior to infection MCMV-Δm157 as described above. After at least 60 days post infection, mice were sacrificed and Maxi CD8 T cells were separated into T_CM_ (CD62L^high^ IL7Rα^high^, cells isolated from pooled lymph nodes and spleen) and T_EM_ (CD62L^low^IL7Rα^low^, cells isolated from the lungs) using a BD FACSAria^TM^ sorter. Sorted populations of cells were labeled with 0.5 µM CFSE (Invitrogen, Basel, Switzerland) for 6 minutes and equal numbers of cells (varying inter-experimentally between 0.5×10^5^ to 1×10^5^) were transferred into latently infected mice or naïve mice. CFSE dilution analysis was assessed 30 days later in the lymph nodes and in the lungs.

### In vitro CD8 T cell proliferation assay

Mini- and Maxi- CD8 T cells were obtained from splenocytes by MACS positive selection with anti-CD8α microbeads. Cells were labeled with 0.5 µM CFSE for 6 minutes. DCs were isolated from the spleen of naïve C57BL/6 mice by MACS positive selection with anti-CD11c microbeads. 1×10^4^ DCs were loaded with serial dilutions of the M38 peptide epitope and were co-cultured with 6×10^5^ Mini- or 6×10^4^ Maxi-CD8 T cells. At day 3 after stimulation, proliferation was measured by CFSE dilution by flow cytometry.

### Quantification of MCMV genomes by qPCR

Spleen and lungs were isolated from mice which have been infected with MCMV-Δm157 for the indicated time, and single cell suspensions were obtained as described in [57], except that no Percoll gradient was performed. Hematopoietic and non-hematopoietic cell populations were sorted based on the expression of CD45. Total genomic DNA was isolated using the Biophenol/Chloroform/Isoamyl alcohol 25:24:1 reagent (Biosolve Ltd, Valkenswaard, Netherlands) and isopropanol-based DNA precipitation. For qPCR, SYBR green incorporation (Qiagen, Hombrechtikon, Switzerland) was measured using a Rotorgene 3000 machine (Corbett Research, Eight Miles Plains, Australia). The reaction was carried out using 100 ng of genomic DNA and 5 µM of primers specific for β-actin (fwd: 5′-CCC TGA AGT ACC CCA TTG AAC-3′, rev: 5′-CTT TTC ACG GTT GGC CTT AG-3′) or M38 (fwd: 5′-AGTCCAGGGTGAGGTCTATG-3′, rev: 5′-CCTTCAACTCGGTGCGATTC-3′). The relative amount of MCMV genome was calculated using the delta-delta Ct method. Results from different runs were normalized using an internal β-actin standard.

## Supporting Information

Figure S1 **M38-specifc CD8 T cell populations in various lymph nodes.** Mediastinal, axillary, cervical and inguinal lymph nodes were pooled from three individual C57BL/6 mice which had been infected with MCMV-Δm157 for 80 days. Representative plots showing the percentages of M38-specific CD8 T cells among total lymphocytes are shown for each lymph node (upper plots), and the lower plots show the expression of KLRG-1 and IL7Rα on M38-specific CD8 T cells. One of two independent experiments is shown.
(PDF)

Figure S2 **Phenotype of Mini and Maxi CD8 T cells.** 10^5^ Mini and 10^4^ Maxi CD8 T cells were adoptively transferred into C57BL/6 mice one day prior to infection, and blood samples were collected at the indicated time points after infection with MCMV-Δm157. Representative plots showing the expression of CD62L and IL7Rα gated on endogenous (CD45.2) and on transferred M38-specific CD8 T cells (CD45.1).
(PDF)

Figure S3 **Priming of MCMV-specific CD8 T cell responses in DC-MHCI chimeric mice.** WT→WT, DC-MHCI→DC-MHCI, WT→DC-MHCI, DC-MHCI→WT chimeric mice were generated by reconstituting WT and DC-MHCI irradiated mice with WT and DC-MHCI bone marrow. At least 6 weeks after reconstitution, mice were infected with MCMV-Δm157 and the M38-and M45-specific CD8 T cells responses were measured from blood samples on day 7 post infection. One of two independent experiments is shown.
(PDF)

Figure S4 **Bcl-2 expression kinetics in M38- and M45-specifc CD8 T cells.** (A)10^5^ Mini CD8 T cells were transferred in WT→WT and WT→H-2 Kb^−/−^ chimeric mice one day prior to infection with MCMV-Δm157. Blood samples were collected on days 7, 9, 11, 13, 17, 21, 27, 42 and 60 post infection. Graphs show the Bcl-2 expression kinetics of M38- (triangles) and M45-specific (circles) CD8 T cells in WT→WT (left graph) and WT→H-2 Kb^−/−^ (right graph) mice. One of two independent experiments is shown. (B) In a separate experiment, Bcl-2 expression was analyzed for M38- and M45-specific CD8 T cells in the lungs of mice which had been infected with MCMV-Δm157 for 7, 14, 28 and 150 days. One of two independent experiments is shown.
(PDF)
