# Supplementary figures and images for: Non-Hematopoietic Cells in Lymph Nodes Drive Memory CD8 T Cell Inflation during Murine Cytomegalovirus Infection
Source: PLoS Pathog. 2011 Oct 27;7(10):e1002313. doi: 10.1371/journal.ppat.1002313 (PMC3203160; doi:10.1371/journal.ppat.1002313)

Fig. S1

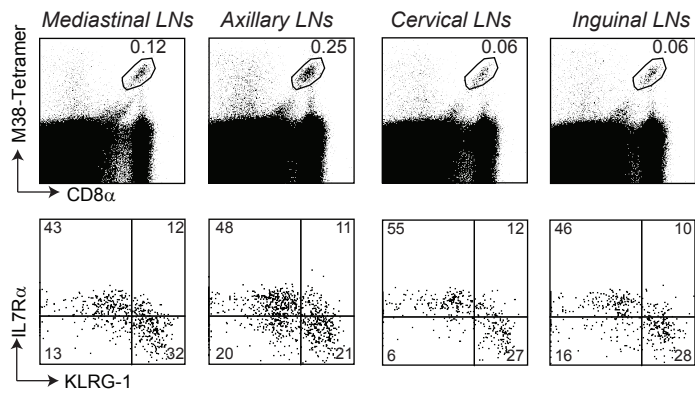

Supplement: Figure S1 — M38-specifc CD8 T cell populations in various lymph nodes. Mediastinal, axillary, cervical and inguinal lymph nodes were pooled from three individual C57BL/6 mice which had been infected with MCMV-Δm157 for 80 days. Representative plots showing the percentages of M38-specific CD8 T cells among total lymphocytes are shown for each lymph node (upper plots), and the lower plots show the expression of KLRG-1 and IL7Rα on M38-specific CD8 T cells. One of two independent experiments is shown. (PDF) [file ppat.1002313.s001.pdf]

Fig. S2

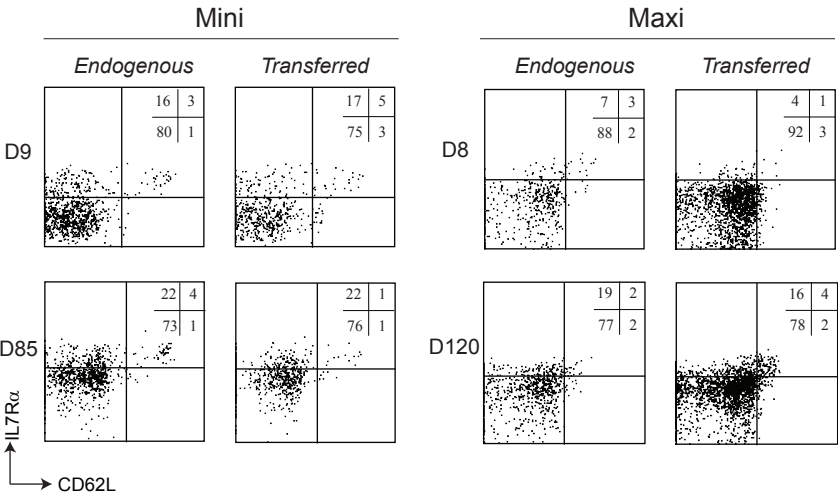

Supplement: Figure S2 — Phenotype of Mini and Maxi CD8 T cells. 105 Mini and 104 Maxi CD8 T cells were adoptively transferred into C57BL/6 mice one day prior to infection, and blood samples were collected at the indicated time points after infection with MCMV-Δm157. Representative plots showing the expression of CD62L and IL7Rα gated on endogenous (CD45.2) and on transferred M38-specific CD8 T cells (CD45.1). (PDF) [file ppat.1002313.s002.pdf]

Fig. S3

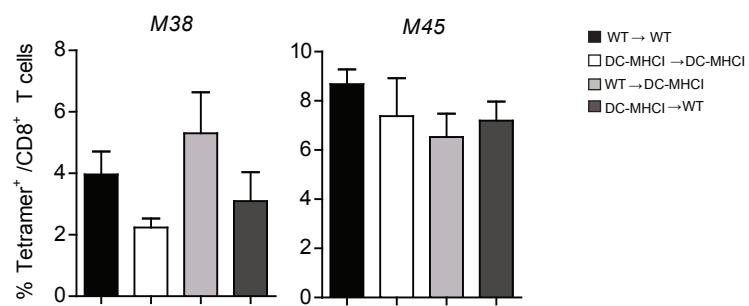

Supplement: Figure S3 — Priming of MCMV-specific CD8 T cell responses in DC-MHCI chimeric mice. WT→WT, DC-MHCI→DC-MHCI, WT→DC-MHCI, DC-MHCI→WT chimeric mice were generated by reconstituting WT and DC-MHCI irradiated mice with WT and DC-MHCI bone marrow. At least 6 weeks after reconstitution, mice were infected with MCMV-Δm157 and the M38-and M45-specific CD8 T cells responses were measured from blood samples on day 7 post infection. One of two independent experiments is shown. (PDF) [file ppat.1002313.s003.pdf]

Fig. S4

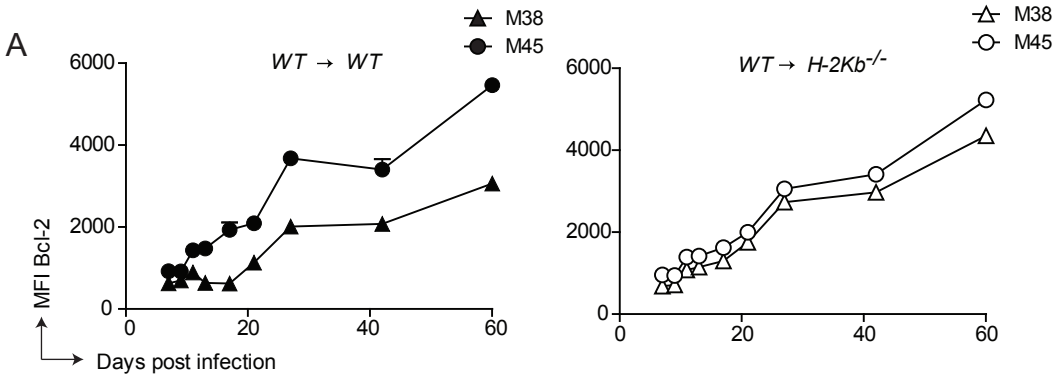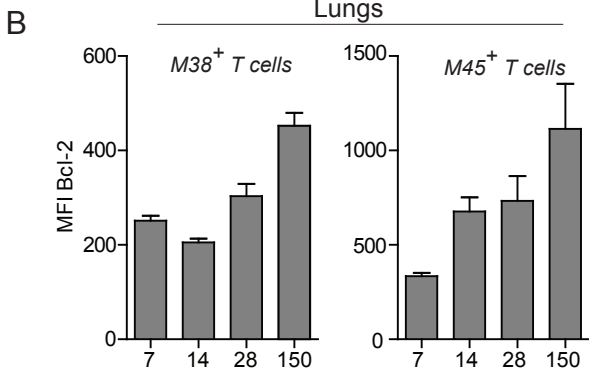

Supplement: Figure S4 — Bcl-2 expression kinetics in M38- and M45-specifc CD8 T cells. (A)105 Mini CD8 T cells were transferred in WT→WT and WT→H-2 Kb−/− chimeric mice one day prior to infection with MCMV-Δm157. Blood samples were collected on days 7, 9, 11, 13, 17, 21, 27, 42 and 60 post infection. Graphs show the Bcl-2 expression kinetics of M38- (triangles) and M45-specific (circles) CD8 T cells in WT→WT (left graph) and WT→H-2 Kb−/− (right graph) mice. One of two independent experiments is shown. (B) In a separate experiment, Bcl-2 expression was analyzed for M38- and M45-specific CD8 T cells in the lungs of mice which had been infected with MCMV-Δm157 for 7, 14, 28 and 150 days. One of two independent experiments is shown. (PDF) [file ppat.1002313.s004.pdf]
